# Metabolomic Profiling of Infectious Parapneumonic Effusions Reveals Biomarkers for Guiding Management of Children with *Streptococcus pneumoniae* Pneumonia

**DOI:** 10.1038/srep24930

**Published:** 2016-04-22

**Authors:** Chih-Yung Chiu, Gigin Lin, Mei-Ling Cheng, Meng-Han Chiang, Ming-Han Tsai, Shen-Hao Lai, Kin-Sun Wong, Sen-Yung Hsieh

**Affiliations:** 1 Department of Pediatrics, Chang Gung Memorial Hospital at Keelung, and Chang Gung University, Taoyuan, Taiwan; 2 Graduate Institute of Clinical Medical Sciences, College of Medicine, Chang Gung University, Taoyuan, Taiwan; 3 Department of Pediatrics, Chang Gung Memorial Hospital at Linkou, and Chang Gung University, Taoyuan, Taiwan; 4 Department of Medical Imaging and Intervention, Chang Gung Memorial Hospital at Linkou, and Chang Gung University, Taoyuan, Taiwan; 5 Graduate Institute of Medical Biotechnology, Chang Gung University, Taoyuan, Taiwan; 6 Department of Clinical Proteomics Center, Chang Gung Memorial Hospital, College of Medicine, Chang Gung University, Taoyuan, Taiwan

## Abstract

Metabolic markers in biofluids represent an attractive tool for guiding clinical management. The aim of this study was to identify metabolic mechanisms during the progress of pleural infection in children with *Streptococcus pneumoniae* pneumonia. Forty children diagnosed with pneumococcal pneumonia were enrolled and analysis of pleural fluid metabolites categorized by complicated parapneumonic effusions (CPE) and non-CPE was assessed by using ^1^H-NMR spectroscopy. Multivariate statistical analysis including principal components analysis (PCA) and partial least-squares discriminant analysis (PLS-DA) were performed. Metabolites identified were studied in relation to subsequent intervention procedures by receiver operating characteristic (ROC) curve analysis. Ten metabolites significantly different between CPE and non-CPE were identified. A significantly lower level of glucose for glycolysis was found in CPE compared to non-CPE. Six metabolites involving bacterial biosynthesis and three metabolites involving bacterial fermentation were significantly higher in CPE compared to non-CPE. Glucose and 3-hydroxybutyric acid were the metabolites found to be useful in discriminating from receiving intervention procedures. Metabolic profiling of pleural fluid using ^1^H-NMR spectroscopy provides direct observation of bacterial metabolism in the progress of pneumococcal pneumonia. An increase in the metabolism of butyric acid fermentation of glucose could potentially lead to the need of aggressive pleural drainage.

Pneumococcal pneumonia is a lung infection caused by *Streptococcus pneumoniae* and is the most common cause of community-acquired pneumonia (CAP)^1^. More than 40% of patients with bacterial pneumonia and 60% of patients with pneumococcal pneumonia develop parapneumonic effusions^2^. Infectious parapneumonic effusion accumulation is posited to be a continuing process of pleural inflammation resulting from the inflammatory response caused by pneumonia. Failing adequate therapy to control the pleural inflammation may progress from simple exudative effusion to empyema formation, requiring aggressive interventions^3^,^4^. An improved understanding the inflammatory process in the accumulated pleural fluid may provide a potential strategy for clinical management.

Biochemical analysis of infectious pleural fluid plays an important role in the management of parapneumonic effusions. Complicated parapneumonic effusions (CPE) are indicated by acidosis (pH ≤ 7.2) associated with raised lactate dehydrogenase (LDH ≥ 1000 U/L) and low glucose levels (glucose ≤ 40 mg/dL) and are more likely to require intervention procedures^5^,^6^. Despite this, proteins in pleural fluids originating from circulation or releasing locally from inflammatory or epithelial cells could also potentially be useful as markers of CPE for guiding clinical management^7^,^8^. A recent study in our laboratory has shown that a diagnostic model construction comprising three down-regulated biomarkers provides an alternative approach for discriminating CPE and subsequent surgical interventions^9^. The identification of a wide range of pleural fluid proteins related to inflammatory progress as biomarkers however is still challenging.

Inflammation in pleural fluids is characterized by a wide variety of cellular and molecular mediators resulting in a broad spectrum of possible metabolic products. Metabolomics using nuclear magnetic resonance (NMR) spectroscopy provides the advantage of studying a wide range of metabolites simultaneously and enables the discovery of small molecule metabolites by revealing any specific biomarker in human disease^10^,^11^. Metabolic markers in biofluids reflect alterations of metabolic fluxes of various organs and cells. Several studies have been reported to reveal metabolites in pleural fluids as biomarkers for exudative pleural effusions including lung cancer and pulmonary tuberculosis^11^,^12^. To date, there are, however, no studies addressing the metabolic changes in the host-microbe interaction of *S. pneumoniae* using infectious parapneumonic effusions.

The major aim of this study was to identify the metabolic profiles of infectious parapneumonic effusions by using ^1^H-NMR spectroscopy in children with pneumococcal pneumonia. The changes in pleural metabolites varies with inflammatory progress were assessed, and their relationships with subsequent intervention procedures were also examined.

## Results

### Population Characteristics

Forty children with pneumococcal pneumonia followed by parapneumonic effusions were consecutively enrolled into our study over a 4-year period. The mean age was 4.3 ± 2.7 years. The pleural fluid met the criteria of CPE (a pH ≤ 7.2, a LDH ≥ 1000 U/L and a glucose ≤40 mg/dL) were seen in 18 (45%) patients; whereas the fluid met the criteria of non-CPE were seen in 22 (55%) patients. The comparisons of pleural variables and characteristics of 40 children with pneumococcal pneumonia are shown in Table 1. A statistically significant higher rate of the identification of *S. pneumoniase* in pleural fluid (83% vs. 41%, *P* = 0.006) and the need of intervention procedures (100% vs. 32%, *P* < 0.001) were found in children with CPE compared to children with non-CPE.

### Identification of Metabolite Sets between CPE and Non-CPE

**Figure 1:**
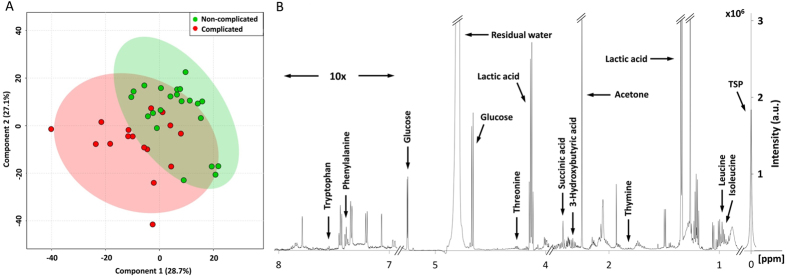
PLS-DA score plots from the analysis of ^1^H-NMR spectra using pleural effusion samples. (**A**) Two-dimensional scatter plot displays the model’s degree of separation between complicated parapneumonic effusions (CPE) and non-CPE. x axis, component 1 (% of total variance); y axis, component 2 (% of total variance) (**B**) Representative 600 MHz ^1^H-NMR spectra of pleural fluid showing the 10 metabolites with VIP scores greater than 1.0 with a *P* value < 0.05 by Mann-Whitney test (δ 0–8). x axis, parts per million (ppm); y axis, intensity (a.u.).

**Figure 2:**
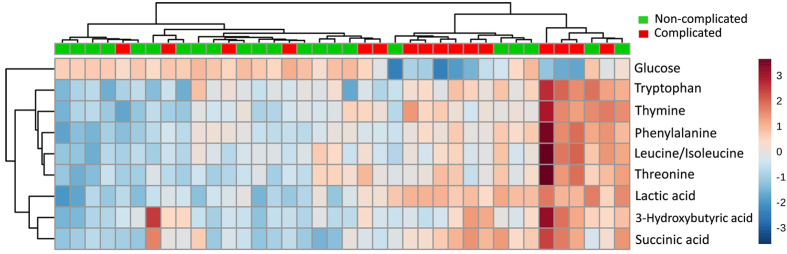
Heat map of 10 metabolites significantly differentially expressed between CPE and non-CPE. Each column represents a pleural fluid sample and each row represents the expression profile of a metabolite. The fold changes from the overall mean concentration are shown in a color-coded way. Blue color represents a decrease, and red color an increase.

^1^H-NMR data of pleural fluid samples obtained were collected and analyzed. One thousand buckets varied across NMR spectra, 184 buckets of which corresponded to 25 of known metabolites (Supplementary Table S1). Unsupervised principal components analysis followed by examination of the first three principal components failed to reveal any clear separation between CPE and non-CPE. Metabolites contributing to the discrimination between CPE and non-CPE were identified by using supervised PLS-DA (Q^2^ = 0.15; R^2^ = 0.32). Metabolites selected by using the cutoff of PLS-DA VIP score >1.0 with a *P* value < 0.05 in the fold change of expression level between CPE and non-CPE are shown in Table 2. A more than 1.4-fold change in these metabolites was observed and volcano plot analysis was used to characterize the differential expressing metabolites between CPE and non-CPE (Supplementary Fig. S1A). These metabolites were further identified by comparison with reference spectra from the Human Metabolome Database (HMDB) and represented in a 2D ^1^H-^13^C NMR spectrum (Supplementary Fig. S1B). The two-dimensional graphs of PLS-DA score plots between CPE and non-CPE and a representative 600 MHz ^1^H-NMR spectra of selected metabolite signals are shown in Fig. 1. Figure 2 shows a heat map of these metabolites clustered using Hierarchical Clustering in the MetaboAnalyst 3.0. Among them, glucose was found to be significantly lower in CPE compared to non-CPE. In contrast, nine metabolites including tryptophan, thymine, phenylalanine, leucine/isoleucine and threonine in cluster 1, and lactic acid, 3-hydroxybutyric acid and succinic acid in cluster 2 were significantly higher in CPE compared to non-CPE.

### Metabolic Pathway and Function Analysis

**Figure 3:**
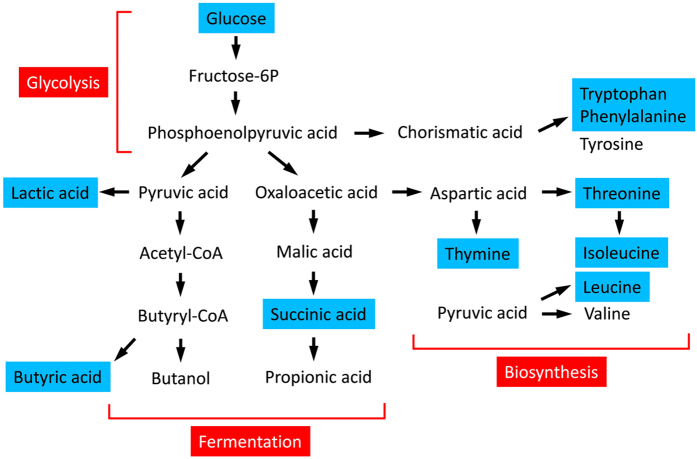
Schematic overview of metabolic pathways in the parapneumonic effusions caused by *Streptococcus pneumonia*. Glucose dissimilation proceeds via glycolysis and leads to fermentation and biosynthesis. Metabolites significantly differentially expressed between CPE and non-CPE are shown in blue color.

Metabolic pathway analysis with MetPA by filtering the dataset using a FDR-adjusted *P* value < 0.05 revealed that significantly different metabolites between CPE and non-CPE were important for amino acid and carbohydrate metabolisms (Table 3). Metabolites in cluster 1 were significantly associated with the biosynthesis of aminoacyl-tRNA and amino acids. Metabolites in cluster 2 were significantly related to carbohydrate metabolism responding for propanoate and butanoate metabolisms. Glucose in cluster 3 was predominantly associated with glycolysis or gluconeogenesis pathway (raw *P* < 0.05). Glucose dissimilation appeared to proceed via glycolysis and led to butyric acid and propionic acid fermentations, and biosynthesis of pyrimidines and amino acids. Figure 3 shows the metabolic pathways of metabolites significantly differentially expressed between CPE and non-CPE.

### Metabolites for Discriminating CPE from Non-CPE and Subsequent Intervention Procedures

**Figure 4:**
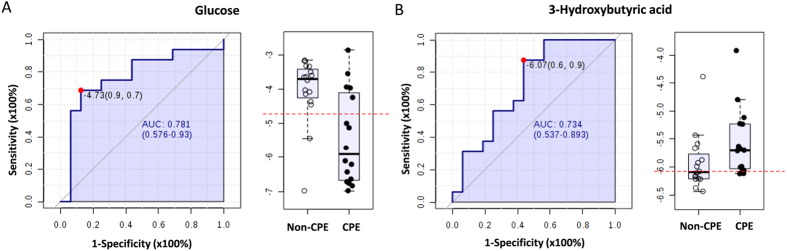
Areas under the ROC curves for discriminating from subsequent intervention procedures. Glucose (**A**); 3-Hydroxybutyric acid (**B**). AUC indicates the area under the curve and the dot refers to the cutoff value maximising sensitivity and specificity for the given samples. Box plots showing median and interquartile ranges of log transformed NMR intensity of metabolites over citric acid by subject groups.

Table 4 shows the analysis of metabolites for discriminating CPE from non-CPE using receiver operating characteristic (ROC) curve analysis. Seven metabolites including glucose, lactic acid, succinic acid, 3-hydroxybutyric acid, thymine, threonine and tryptophan had the highest AUC significantly different from 0.5 (*P* < 0.05). After echo-guided thoracentesis, pleural metabolites of patients whose fever subsided (n = 15) and patients who received intervention procedures (n = 18) within 48 hours were compared and analyzed. Glucose (AUC = 0.781; 95% CI: 0.576–0.930; *P* = 0.008) and 3-hydroxybutyric acid (AUC = 0.734; 95% CI: 0.537–0.893; *P* = 0.032) were the metabolites found to be useful in discriminating from subsequent intervention procedures (Fig. 4).

## Discussion

Infectious parapneumonic effusion is excess fluid that accumulates in the pleural cavity as a result of infections when lung tissues are damaged. With the progress of infection and inflammation in pleural fluids, metabolic changes in glucose associated with acidosis occur and require drainage for resolution. This study provides an overview of metabolic changes occurring in the infectious parapneumonic effusions and reveals potential metabolites for guiding management of infectious parapneumonic effusions in children with *Streptococcus pneumonia* pneumonia.

Complicated parapneumonic effusions (CPE) and empyema are well recognized complications in children with pneumococcal pneumonia^13^. Biochemical analysis of infectious pleural fluid, including pH, glucose, and LDH concentrations can significantly discriminate complicated from non-complicated parapneumonic effusions as in this study. In addition, the detection rate of *S. pneumonia* and number of neutrophils were found to be significantly higher in CPE compared to non-CPE. These findings indicate that CPE occur as a result of bacterial invasion into the pleural space that leads to an increased number of neutrophils and a series of metabolic processes.

*Streptococcus pneumoniae* is a Gram-positive diplococcus with a well-formed capsule and is recognized as a major cause of community-acquired pneumonia in children. The growth of pneumococci in the host depends on the catabolism of utilized carbon sources. The Embden-Meyerhof-Parnas (EMP) pathway for glucose dissimilation leading to pyruvate is the pathway at the center of metabolism in *S. pneumonia*^14^,^15^. The ^13^C isotopologue patterns in amino acids from labelled glucose have revealed the pathways to the *de novo* synthesized amino acids in *S. pneumonia*^16^. In this study, amino acids significantly increased during the progressive pleural inflammation were involved in the pathways to amino acid biosynthesis in *S. pneumoniae*, indicating that an increased pneumococcal invasion in CPE may lead to the aggressive metabolic processes of glucose consumption and subsequent biosynthesis of amino acids.

*Streptococcus pneumoniae* is a facultative anaerobe and fermentative microorganism. A facultative anaerobe is an organism that makes ATP by aerobic respiration if oxygen is present, but is capable of switching to fermentation or anaerobic respiration if oxygen is absent^17^. Under hypoxic conditions, pyruvate is mainly converted by LDH to lactic acid. Several studies have indicated that bacterial and cellular metabolism in pleural fluids cause the consumption of glucose and the excretion of lactate as in this study^4^,^18^,^19^. Increased propanoate and butanoate metabolisms along with significantly higher levels of succinic acid and 3-hydroxybutyric acid were also seen in CPE in this study. The anaerobic metabolic pathways for lactic acid, propionic and butyric acid fermentation of glucose leading to the excretion of protons and carbon dioxide (CO_2_) accumulation may result in a low pleural fluid glucose and pH.

Aggressive pleural drainage should be performed when the progression of CPE is not controlled and pleural fluid pH has been reported to be the best biochemical aid in predicting of CPE^20^. In this study, glucose and 3-hydroxybutyric acid were the metabolites useful for discriminating from CPE and subsequent intervention procedures, indicating that an increase in the metabolism of butyric acid fermentation of glucose with pleural fluid acidosis plays an important role in guiding management of infectious parapneumonic effusions caused by *S. pneumonia*. However, butyric acid is produced as end-product of a fermentation process solely performed by obligate anaerobic bacteria such as *Clostridium* spp^21^. Clinically, anaerobic bacteria contribute significantly to pleural infection, being identified as the sole or co-pathogen in 25–76% of pediatric cases^22^. In children with pneumococcal pneumonia, antibiotic choices should be informed by the results of blood and pleural fluid cultures and sensitivities; an anaerobic antibiotic choice may be considered for children with uncontrolled CPE and empyema.

A limitation of this study is the small sample size and a relatively low sensitivity of NMR-based metabolomic analysis than mass spectrometry-based methods. However, a combination of NMR and PLS-DA provides the advantage of studying a wide range of metabolites associated with bacterial and cellular pathways with accuracy. The overall comparability of pleural samples from non-CPE to CPE may result in the low R^2^ and Q^2^ scores in this study, further studies with a larger sample size with subgroup analysis by reducing within group variability may be needed to investigate more comprehensively. Despite this, a significant strength of the present study lies in its short time interval during which there were no major changes in surgical strategy for CPE. An age-matched comparison of children with CPE and non-CPE also makes the results demonstrated here are valid and potentially important.

In conclusion, metabolic profiling of pleural fluid using ^1^H-NMR spectroscopy provides new insight into the role of bacterial metabolism and physiology in the progress of pneumococcal pneumonia, and reveals putative biomarkers for requiring aggressive pleural drainage. An increased pneumococcal invasion in CPE may increase metabolic process of glucose consumption along with amino acid biosynthesis. A significantly increase in butyric acid fermentation of glucose in children with CPE receiving intervention procedures suggests that a mixed infection of anaerobic bacteria may contribute to the progress of pleural infection and antibiotics with anaerobic coverage should be considered to avoid further morbidity or mortality.

## Methods

### Study Population

The study population consisted of children who had pneumococcal pneumonia complicated by parapneumonic effusions requiring hospitalization. *Streptococcus pneumoniae* infection was defined by a positive result in blood or pleural fluid culture or the detection of antigens in the pleural fluid by latex agglutination testing^23^. Acute pneumococcal infection was also included for patients who had necrotic lung parenchyma with a positive urine test for *S. pneumoniae* (Binax, Portland, ME, USA). In general practice, antibiotic selection covered the likeliest organisms and adjusted after culture results were obtained. Intervention procedures for intercostal drainage including tube thoracostomy or video-assisted thoracoscopic surgery (VATS) were considered if patients had persistent fever >39 °C, dyspnea, and sepsis despite appropriate antibiotic therapy^24^. This study was approved by the Ethic Committee of Chang Gung Memory Hospital (No. 93-6299). All experiments in this study were performed in accordance with the relevant guidelines and regulations and written informed consent was obtained from the parents or guardians of all study subjects.

### Pleural Fluid Collection

All subjects underwent a standard thoracocentesis procedure and pleural effusion samples were collected before intervention procedures. Pleural fluid was immediately analyzed for pH, total cell counts and differential cell count, and for glucose and LDH concentrations. Complicated parapneumonic effusions (CPE) were diagnosed if the pleural fluid met at least two of the following criteria: a pleural fluid pH of ≤7.2, a LDH level of ≥1000 U/L, and a glucose level of ≤40 mg/dL^4^,^9^. Otherwise, non-complicated parapneumonic effusions (non-CPE) were defined as patients whose pleural fluid met only one or none of the above criteria. Another 4 milliliters of specimen, if available, were mixed in Greiner Bio-One VACUETTE Coagulation Tubes with 3.2% sodium citrate anticoagulant solution (1 mL) in a ratio of 9:1 pleural fluid to citrate, which were immediately immersed in ice separately and centrifuged at 1,500 g for 10 min. The cell-free supernatant from each sample was stored at −80 °C until further use.

### Sample Preparation

Pleural fluid samples collected from children with pneumococcal pneumonia were selected and examined. After thawing, to stabilize the pH value across samples prior to spectrum acquisition, 500 μL of pleural effusion was mixed with 250 μL of phosphate buffer (0.075 M Na_2_HPO_4_, pH 7.4) in deuterium water which containing 0.08% 3-(trimethylsilyl)-propionic-2,2,3,3-d_4_ acid sodium salt (TSP) as an internal chemical shift reference standard, 2 mM NaN_3_ as an inhibitor of bacterial contamination. Each sample was vortexed for 20 s and subsequently centrifuged at 12000 g at 4 °C for 30 min. After centrifugation, a 600 μL aliquot of the supernatant was transferred to a standard 5 mm NMR tube for analysis.

### ^1^H–Nuclear Magnetic Resonance (NMR) Spectroscopy

^1^H-NMR spectra were acquired on a Bruker Avance 600 MHz spectrometer (Bruker-Biospin GmbH, Karlsruhe, Germany) equipped with a 5 mm CPTCI ^1^H cryoprobe at Chang Gung Healthy Aging Research Center, Taiwan. Temperature was controlled at 300 K throughout the experiments. Low-power water pre-saturation pulse sequence was used for water signal suppression during the relaxation time of 4 s. For each spectrum, 64 scans were collected into 64K computer data points using a spectral width of 10,000 Hz (10 ppm). All 1D spectra were applied for analysis before Fourier transformation with zero-filled to exponential line-broadenings of 0.3 Hz. The acquired ^1^H-NMR spectra were manually phased, baseline-corrected, and referenced to the chemical shift of TSP (δ 0.0 ppm) using TopSpin 3.2 software (Bruker BioSpin, Rheinstetten, Germany).

### NMR Data Processing and Analysis

The raw ^1^H-NMR spectra were imported into AMIX version 3.9.12 (Bruker BioSpin, Rheinstetten, Germany) for spectral bucket, spectral region exclusion and spectral normalization. ^1^H-NMR spectra were aligned on the TSP peak and normalized on the spectral area for calculating the concentration of each metabolite in the spectral peaks of each metabolite. The ^1^H-NMR spectra were subdivided into integrated regions of 0.01 ppm corresponding to the region of δ 0–10 ppm. Regions containing residual water (δ 4.825–4.725 ppm) were excluded from the data set to avoid spectral interference of residual water. Additional optimization of ^1^H-NMR spectra normalization was checked by using biochemical glucose concentration. The area of individual resonances of glucose metabolite was not significantly correlated with biochemical glucose concentration but the ratio of glucose/citric acid was (Supplementary Fig. S2). The spectra were therefore normalized to the integral of citric acid peak at δ 2.575–2.525 ppm to overcome the variation of pleural fluid amount when collecting samples. All metabolites in pleural fluid were identified using Chenomx NMR Suite 8.1 professional software (Chenomx Inc., Edmonton AB, Canada) with full resolution NMR data. A standard two-dimensional (2D) NMR experiment was conducted on a pooled pleural sample and metabolites were further assigned by comparison with reference spectra from the Human Metabolome Database (HMDB). For identifying metabolites contributing to the discrimination between groups, the normalized ^1^H-NMR bucket data were uploaded to MetaboAnalyst 3.0 (http://www.metaboanalyst.ca) for partial least squares-discriminant analysis (PLS-DA). All the NMR spectra were generalized log transformed (glog), method offered in MetaboAnalyst, to stabilize the variance across the spectral bins and to increase the weightings of the less intense peaks^25^. The spectral variables were mean-centered and scaled to unit variance, and 10-fold internal cross-validation was performed to evaluate the quality of the resulting statistical models by considering the diagnostic measures R^2^ and Q^2^ 26. Potential metabolites were selected based on the Variable Importance in Projection (VIP) score greater than 1.0. The functional pathway analysis of potential biomarkers was based on the database source of the Kyoto Encyclopedia of Genes and Genomes (http://www.genome.jp/kegg/). Receiver Operating Characteristic (ROC) analysis of entire dataset was employed to measure the strength of metabolites for identifying potential biomarkers. The ROC curve analysis was performed using the online ROCCET (ROC curve explorer and tester, http://www.roccet.ca)^27^.

### Statistical Analysis

Distribution of baseline characteristics and pleural variables of children with pneumococcal pneumonia categorized by CPE and non-CPE was done with univariable parametric and non-parametric tests such as Student’s *t*-test, Mann-Whitney test, χ^2^, and Fisher’s exact test. Hierarchical clustering was performed and heat maps were created based on the Pearson correlation and the Ward clustering algorithm. The fold changes, volcano plots, and statistical significance in metabolites between CPE and non-CPE were performed by the non-parametric Mann-Whitney test using MetaboAnalyst web server. Statistical analysis was performed by using the Statistical Program for Social Sciences (IBM SPSS Statistics for Windows, Version 20.0; IBM, Armonk, NY, USA). All statistical hypothesis tests were two tailed and a *P* value of less than 0.05 was considered to be significant.

## Additional Information

**How to cite this article**: Chiu, C.-Y. *et al*. Metabolomic Profiling of Infectious Parapneumonic Effusions Reveals Biomarkers for Guiding Management of Children with *Streptococcus pneumoniae* Pneumonia. *Sci. Rep.* **6**, 24930; doi: 10.1038/srep24930 (2016).

## Supplementary Material

Supplementary Information

## Figures and Tables

**Table 1:** Comparison of characteristics of patients and pleural variables in complicated and non-complicated parapneumonic effusions.

Characteristics	Non-CPE (n = 22)	CPE (n = 18)	*P*
Age, yr	4.6 ± 3.4	3.9 ± 1.3	0.663
Sex, male	12 (55)	8 (44)	0.525
Days of fever elapsed prior to thoracentesis	9.6 ± 5.2	8.5 ± 2.9	0.478
*S. pneumoniae* detection			0.006
Blood culture or antigen in pleural fluid	9 (41)	15 (83)	
Urinary pneumococcal antigen	13 (59)	3 (17)	
Hemogram			
WBC, 10^9^/L	13.2 ± 8.4	16.5 ± 7.2	0.192
Hb, g/dL	10.4 ± 1.2	9.9 ± 2.5	0.414
Platelet, 10^9^/L	358.7 ± 246.1	317.7 ± 202.7	0.574
CRP, mg/L	176.3 ± 152.8	223.0 ± 108.7	0.222
Pleural effusion			
WBC, 10^9^/L	3.8 ± 4.1	52.4 ± 89.3	0.048
pH	7.35 ± 0.08	6.94 ± 0.28	0.001
Glucose, mg/dL	72.2 ± 16.4	16.7 ± 23.3	<0.001
LDH, IU/L	605.5 ± 371.9	11,553 ± 10,369	<0.001
Intervention procedures	7 (32)	18 (100)	<0.001
Hospital stay, d	11.9 ± 3.7	14.4 ± 3.2	0.045

Data shown are mean ± SD or number (%) of patients as appropriate.

Abbreviations: CPE, complicated parapneumonic effusions; yr, year; WBC, white blood cell; Hb, hemoglobin; CRP, C-reactive protein; LDH, lactate dehydrogenase; d, day.

**Table 2:** The VIP score and fold change of metabolites significantly differentially expressed between CPE and non-CPE.

Metabolites	Chemical shift, ppm (multiplicity)	VIP score^a^	Fold change^b^	*P* value
Glucose	5.255–5.245 (d)	2.92	0.43	<0.001
Lactic acid	4.145–4.115 (q)	1.62	1.49	0.015
Thymine	1.845 (s)	1.42	1.80	0.021
Succinic acid	2.415 (s)	1.35	1.48	0.016
Tryptophan	7.565–7.535 (d)	1.32	1.61	0.033
3-Hydroxybutyric acid	2.335–2.325 (m)	1.25	1.49	0.020
Phenylalanine	7.405–7.375 (m)	1.12	1.49	0.029
Threonine	4.295–4.245 (m)	1.11	1.49	0.030
Leucine/Isoleucine	0.985–0.935 (t)	1.06	1.55	0.047

Abbreviations: VIP, Variable Importance in Projection; CPE, complicated parapneumonic effusions; ppm, parts per million; d, doublet; s, singlet; m, multiplet; t, triplet; q, quartet.

^a^VIP score was obtained from PLS-DA model.

^b^Fold change was calculated by dividing the value of metabolites in CPE by non-CPE and compared by a non-parametric Mann-Whitney test.

**Table 3:** Metabolic pathway and function analysis between CPE and non-CPE.

Cluster	Metabolites	Pathway Name	Total	Hits	Raw*P*	FDR	Function
1	Leucine, phenylalanine, threonine, isoleucine, tryptophan, thymine	Aminoacyl-tRNA biosynthesis	75	5	1.51E-07	1.21E-05	Genetic Information Processing; Translation
		Valine, leucine and isoleucine biosynthesis	27	3	2.46E-05	9.86E-04	Amino acid metabolism
		Phenylalanine, tyrosine and tryptophan biosynthesis	27	2	1.77E-03	4.72E-02	Amino acid metabolism
2	3-Hydroxybutyric acid, lactic acid, succinic acid	Propanoate metabolism	35	2	6.11E-04	3.20E-02	Carbohydrate metabolism
		Butanoate metabolism	40	2	8.00E-04	3.20E-02	Carbohydrate metabolism
3	Glucose	Glycolysis or Gluconeogenesis	31	1	1.29E-02	4.16E-01	Carbohydrate metabolism

Total is the total number of compounds in the pathway; the Hits is the actually matched number from the user uploaded data; the Raw *P* is the original *P* value calculated from the enrichment analysis; the FDR is the portion of false positives above the user-specified score threshold.

Abbreviations: CPE, complicated parapneumonic effusions; FDR, false discovery rate.

**Table 4:** Areas under the ROC curve of metabolites for discriminating CPE and subsequent intervention procedures.

Metabolites	CPE			Intervention procedures		
	AUC	95% CI	*P* value^a^	AUC	95% CI	*P* value^a^
Glucose	0.824	0.650–0.948	0.001	0.781	0.576–0.930	0.008
Lactic acid	0.739	0.572–0.878	0.013	0.671	0.472–0.885	0.096
Succinic acid	0.743	0.571–0.876	0.013	0.703	0.518–0.888	0.050
3-Hydroxybutyric acid	0.741	0.555–0.862	0.014	0.734	0.537–0.893	0.032
Thymine	0.732	0.545–0.879	0.020	0.684	0.492–0.876	0.076
Threonine	0.707	0.528–0.868	0.041	0.625	0.427–0.823	0.228
Tryptophan	0.695	0.520–0.841	0.044	0.672	0.484–0.859	0.097

Abbreviations: ROC, receiver operating characteristic; CPE, complicated parapneumonic effusions; AUC, area under the curve; CI, confidence index.

^a^*P* value was determined by ROC curve analysis in SPSS.
