# Supplementary material for: Metabolomic Profiling of Infectious Parapneumonic Effusions Reveals Biomarkers for Guiding Management of Children with Streptococcus pneumoniae Pneumonia
Source: Sci Rep. 2016 Apr 22;6:24930. doi: 10.1038/srep24930 (PMC4840347; doi:10.1038/srep24930)
Supplement: Supplementary Information [file srep24930-s1.pdf]

Supplementary information

Metabolomic Analysis of Infectious Parapneumonic Effusions Reveals Biomarkers for Guiding Management of Children with *Streptococcus pneumoniae* Pneumonia

Chih-Yung Chiu, Gigin Lin, Mei-Ling Cheng, Meng-Han Chiang, Ming-Han Tsai, Shen-Hao Lai, Kin-Sun Wong & Sen-Yung Hsieh

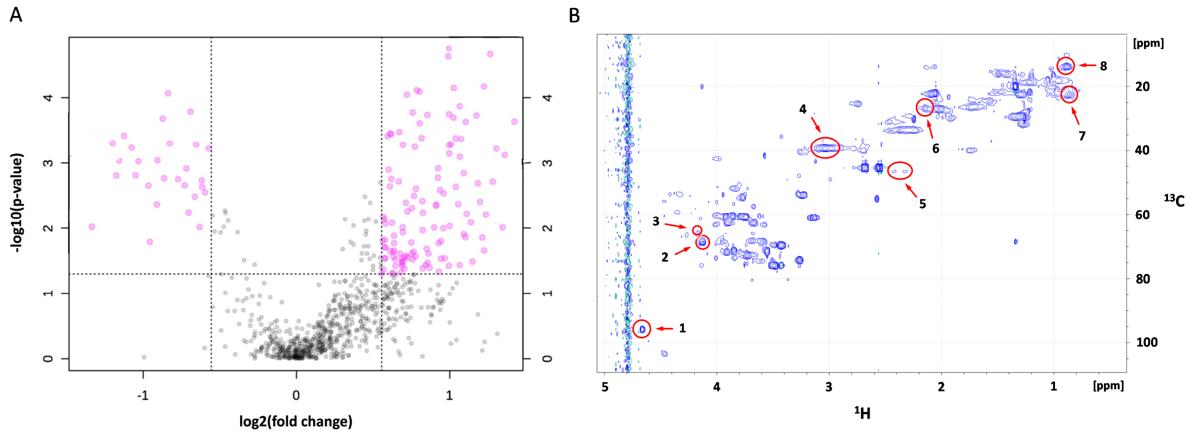

Supplementary Figure S1. Differential expressing metabolites representing in a volcano plot and a 2D NMR spectrum (^{1}H and ^{13}C NMR spectrum). (A) Volcano plot analysis illustrated of metabolites between CPE and non-CPE. Pink circle represents the metabolite displayed with a significance threshold of *p*-value < 0.05 and fold change > 1.4. x axis, log of the fold change; y axis, negative log of the *p*-value. (B) A representative 2D ^{1}H-^{13}C heteronuclear single quantum coherence (HSQC) spectrum showed ^{1}H connected to a ^{13}C. Differential metabolites were identified using Chenomx NMR Suite 8.1 and further assigned by comparison with reference spectra from the Human Metabolome Database (HMDB). x axis, ^{1}H spectrum; y axis, ^{13}C spectrum. 1, Glucose; 2, lactic acid; 3, threonine; 4, phenylalanine; 5, 3-hydroxybutyric acid; 6, succinic acid; 7, leucine; 8, isoleucine.

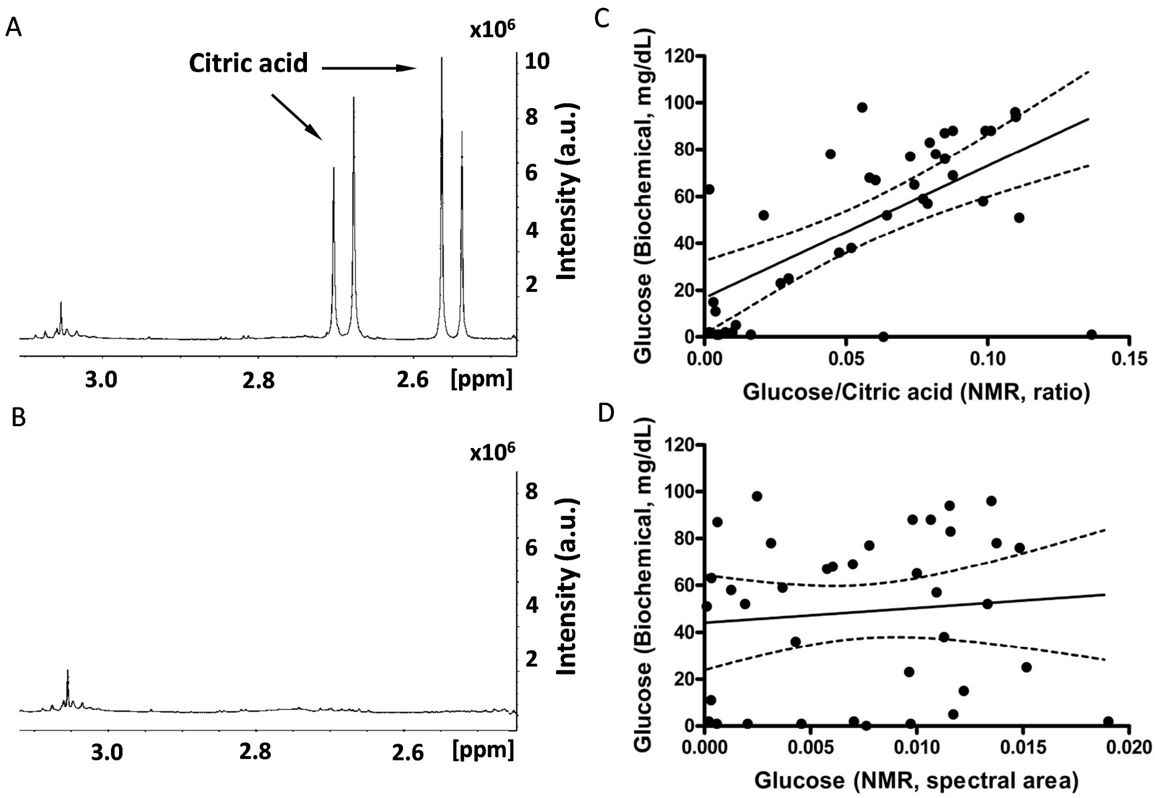

Supplementary Figure S2. Optimization of ^{1}H-NMR spectra normalization procedure. (A) Representative 600 MHz ^{1}H-NMR spectra of pleural fluid obtained from the same individual showed the much higher signal intensity of citric acid having 3.2% sodium citrate anticoagulant solution. (B) Citric acid was difficult to detect in infectious parapneumonic effusion without adding 3.2% sodium citrate. (C) In this study, all ^{1}H-NMR spectra were obtained from pleural effusion with 3.2% sodium citrate and the ratio of glucose/citric acid was significantly correlated with biochemical glucose concentration (Spearman, r = 0.95; $P < 0.001$). (D) The area of individual resonances of glucose metabolite however was not significantly correlated with biochemical glucose concentration (Spearman, r = 0.18; $P = 0.271$).

Supplementary Table S1. ^{1}H-NMR assignment results of the identified metabolites, chemical shifts and VIP scores in pleural effusion samples, and fold changes differentially expressed between CPE and non-CPE.

Compound name	Chemical shift, ppm (multiplicity)	VIP score[a]	Fold change[b]	*P* value
Glucose	5.255-5.245 (d)	2.92	0.43	<0.001
Lactic acid	4.145-4.115 (q)	1.62	1.49	0.015
Thymine	1.845 (s)	1.42	1.80	0.021
Succinic acid	2.415 (s)	1.35	1.48	0.016
Tryptophan	7.565-7.535 (d)	1.32	1.61	0.033
3-Hydroxybutyric acid	2.335-2.325 (m)	1.25	1.49	0.020
Phenylalanine	7.405-7.375 (m)	1.12	1.49	0.029
Threonine	4.295-4.245 (m)	1.11	1.49	0.030
Leucine/Isoleucine	0.985-0.935 (t)	1.06	1.55	0.047
Galactose	4.605-4.595 (t)	1.05	1.65	0.067
Alanine	1.505-1.475 (d)	1.02	1.56	0.081
Valine	1.005-0.995 (d)	0.85	1.25	0.063
Histidine	7.095-7.065 (d)	0.84	1.33	0.092
3-Methy-2-oxovaleric acid	1.125 (d)	0.76	1.24	0.099
Tyrosine	7.215-7.195 (m)	0.75	1.26	0.156
Glutamic acid	2.375-2.355 (m)	0.65	1.19	0.179
Cis-Acotinic acid	5.695-5.675 (s)	0.61	1.50	0.362
Arabinose	4.535-4.515 (d)	0.47	1.37	0.481
Hypoxanthine	8.225-8.195 (s)	0.37	0.98	0.538
Ethanol	1.195-1.185 (t)	0.23	0.90	0.706
Formic acid	8.485-8.455 (s)	0.19	1.04	0.668
Acetone	2.245-2.235 (s)	0.15	0.95	0.820
Citric acid	2.705-2.655 (d)	0.09	1.00	0.214
Fumaric acid	6.545-6.525 (s)	0.01	0.83	0.988

Abbreviations: VIP, Variable Importance in Projection; CPE, complicated parapneumonic effusions; ppm, parts per million; d, doublet; s, singlet; m, multiplet; t, triplet; q, quartet.

[a]VIP score was obtained from PLS-DA model.

[b]Fold change was calculated by dividing the value of metabolites in CPE by non-CPE and compared by a non-parametric Mann-Whitney test.
